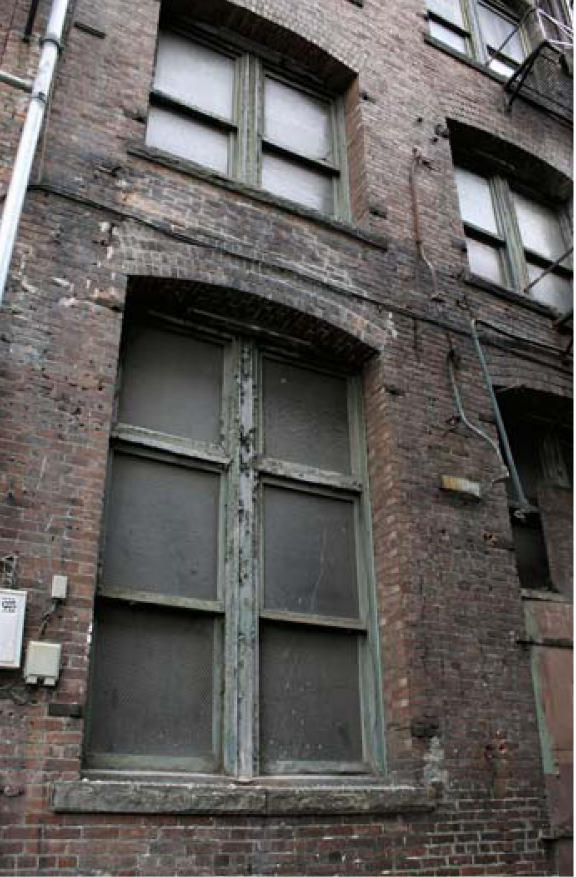# Air Pollution: Urban Grime Recycles Toxics

**Published:** 2007-09

**Authors:** Carol Potera

Grime clinging to windows and other surfaces in cities may be an unrecognized source of toxic nitrogen oxide (NO_x_) pollutants that can damage lung tissue, according to research in the 1 June 2007 issue of *Environmental Science & Technology*. Atmospheric chemist James Donaldson and colleagues at the University of Toronto found that when sunlight strikes grime, inactive nitrogen compounds may be transformed into active forms that become airborne. This view challenges the long-standing notion that nitrogen compounds trapped in grime (also known as urban surface film) are washed away by rain and are no longer available to produce ozone and smog.

The combustion of fossil fuels by automobiles and power plants forms NO_x_, which combines with volatile organic compounds to produce smog. Donaldson and colleagues observed that nitrogen compounds sequestered in urban surface films seemed to disappear faster than could be explained by rain washing them away into soil or groundwater. To test this idea, Donaldson’s team coated slides with a few key chemicals found in grime and exposed them to gaseous nitric acid. This main form of nitrogen is considered to be an atmospheric end product; it sticks to windows, where it is assumed to be inert. When the slides were irradiated with visible light, nitric acid was removed, likely converted to nitrogen dioxide, nitric oxide, and nitrous acid—all major players in ozone and smog formation.

The discovery suggests that “nitrogen oxides are being recycled and are not lost as people have thought,” Donaldson says. He plans to repeat the experiment with actual grime scraped from dirty windows to verify whether sunlight releases NO_x_ from complex natural urban surface films. These films are composed of several broad classes of chemicals, some with hundreds of identified compounds, according to research published in volume 63, issue 1 (2006) of *Chemosphere*. If the initial results hold up, computer models of urban air quality may need to be adjusted to account for this overlooked supply of photochemically activated compounds.

“This is not the first suggestion that nitric acid can be recycled back into photochemically active forms in the gas phase,” says Barbara Finlayson-Pitts, a professor of chemistry at the University of California, Irvine. “Renoxification” reactions were proposed back in the 1990s, and Finlayson-Pitts’s own group has done work in this area. However, she adds, “The idea that chemistry on the surfaces of buildings plays a significant role in the chemistry of the atmosphere has not been given much attention,” and Donaldson’s study nicely illustrates that it could prove to be critically important for developing accurate computer models of air pollution.

## Figures and Tables

**Figure:**